# Blue blood on ice: modulated blood oxygen transport facilitates cold compensation and eurythermy in an Antarctic octopod

**DOI:** 10.1186/s12983-015-0097-x

**Published:** 2015-03-11

**Authors:** Michael Oellermann, Bernhard Lieb, Hans-O Pörtner, Jayson M Semmens, Felix C Mark

**Affiliations:** Alfred-Wegener-Institute Helmholtz Centre for Polar and Marine Research, Am Handelshafen 12, 27570 Bremerhaven, Germany; Institute of Zoology, Johannes Gutenberg-Universität, Müllerweg 6, 55099 Mainz, Germany; Fisheries, Aquaculture and Coasts Centre, Institute for Marine and Antarctic Studies (IMAS), University of Tasmania, Hobart, Tasmania 7001 Australia

**Keywords:** Haemocyanin, Hemocyanin, Cephalopod, Oxygen affinity, Oxygen carrying capacity, Diffusion chamber, *Pareledone charcoti*, *Octopus pallidus*, *Eledone moschata*

## Abstract

**Introduction:**

The Antarctic Ocean hosts a rich and diverse fauna despite inhospitable temperatures close to freezing, which require specialist adaptations to sustain animal activity and various underlying body functions. While oxygen transport has been suggested to be key in setting thermal tolerance in warmer climates, this constraint is relaxed in Antarctic fishes and crustaceans, due to high levels of dissolved oxygen. Less is known about how other Antarctic ectotherms cope with temperatures near zero, particularly the more active invertebrates like the abundant octopods. A continued reliance on the highly specialised blood oxygen transport system of cephalopods may concur with functional constraints at cold temperatures. We therefore analysed the octopod’s central oxygen transport component, the blue blood pigment haemocyanin, to unravel strategies that sustain oxygen supply at cold temperatures.

**Results:**

To identify adaptive compensation of blood oxygen transport in octopods from different climatic regions, we compared haemocyanin oxygen binding properties, oxygen carrying capacities as well as haemolymph protein and ion composition between the Antarctic octopod *Pareledone charcoti*, the South-east Australian *Octopus pallidus* and the Mediterranean *Eledone moschata*. In the Antarctic *Pareledone charcoti* at 0°C, oxygen unloading by haemocyanin was poor but supported by high levels of dissolved oxygen. However, lower oxygen affinity and higher oxygen carrying capacity compared to warm water octopods, still enabled significant contribution of haemocyanin to oxygen transport at 0°C. At warmer temperatures, haemocyanin of *Pareledone charcoti* releases most of the bound oxygen, supporting oxygen supply at 10°C. In warm water octopods, increasing oxygen affinities reduce the ability to release oxygen from haemocyanin at colder temperatures. Though, unlike *Eledone moschata*, *Octopus pallidus* attenuated this increase below 15°C.

**Conclusions:**

Adjustments of haemocyanin physiological function and haemocyanin concentrations but also high dissolved oxygen concentrations support oxygen supply in the Antarctic octopus *Pareledone charcoti* at near freezing temperatures. Increased oxygen supply by haemocyanin at warmer temperatures supports extended warm tolerance and thus eurythermy of *Pareledone charcoti*. Limited haemocyanin function towards colder temperatures in Antarctic and warm water octopods highlights the general role of haemocyanin oxygen transport in constraining cold tolerance in octopods.

**Electronic supplementary material:**

The online version of this article (doi:10.1186/s12983-015-0097-x) contains supplementary material, which is available to authorized users.

## Introduction

The Antarctic Ocean forms an extreme habitat with temperatures ranging between −1.8 to 2°C all year round e.g. [1,2]. Most marine animals living under these conditions are unable to regulate their body temperature (ectotherms) and are thus required to sustain body functions at near freezing temperatures, via numerous adjustments at the molecular, cellular or systemic level [3]. On the other hand, Antarctic waters are rich in oxygen due to increased solubility of oxygen and rigorous mixing across the water column [4]. Paired with low metabolic rates, commonly found among Antarctic ectotherms [5-7], oxygen supply seems less challenging in the cold, as demonstrated by the ability of Antarctic notothenioid fishes to sustain life with low levels of haemoglobin [8] and in case of the Antarctic icefishes (Channichthyidae), even with the complete absence of oxygen transport proteins in the cold [4,9]. Conversely, cold temperatures may hamper oxygen supply by lowered diffusion across tissue and cellular boundaries, increased viscosity [10] and often a decreased ability of blood pigments like vertebrate haemoglobin or cephalopod haemocyanin to release oxygen to tissues as the pigment’s affinity for oxygen increases [11-13]. Antarctic fishes cope with these challenges by increased mitochondrial and membrane densities supporting diffusion [10], loss of blood cells reducing blood viscosity [14] or lowered oxygen affinity sustaining oxygen transport by their haemoglobins [15-17]. Little is known whether Antarctic ectotherms other than fish evolved comparable physiological adaptations to sustain oxygen supply in the cold.

**Figure 1:**
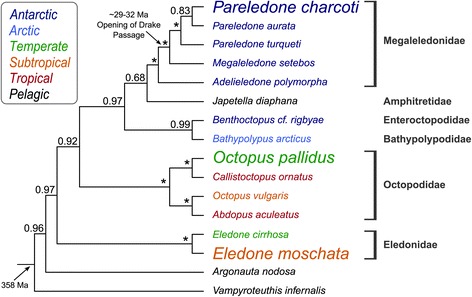
**Phylogenetic relationships of the three octopod species analysed in this study and related octopodiformes.** The Bayesian phylogenetic tree was based on the mitochondrial genes cytochrome oxidase subunit I and III and the nuclear genes rhodopsin, octopine dehydrogenase and 16S rDNA. *Vampyroteuthis infernalis* and *Argonauta nodosa* served as outgroup. Posterior probabilities were shown above nodes with stars marking values of 1.0. Colours denote the climatic origin. The opening of the Drake Passage ca. 29–32 million (Ma) years ago (position marked on tree was taken from [26]), denoting the isolation of Antarctic waters from warmer waters, preceded the diversification of the Antarctic genus *Pareledone. Pareledone charcoti* belongs to the endemic Southern Ocean octopod family Megaleledonidae and shares ancestry with *Adelieledone polymorpha*. This species inhabits the northern Antarctic Peninsula and the Scotia Arc island bridge connecting shallow South American waters with the Antarctic shelf, indicating an origin from temperate shallow waters [27]. *Octopus pallidus* and *Eledone moschata* belong to distinct families of non-polar shallow water octopods [28].

Among those other ectotherms are numerous species of Antarctic octopods, which occur exclusively in the Antarctic Ocean and form an important part of the benthic megafauna as both prey and predators [18-22]. Although their origin is still unclear, Antarctic octopods may have evolved *in situ* in shallow Southern Ocean waters [23], or colonised Antarctic shelves from the deep-sea [24] or possibly in the case of the genera *Pareledone* or *Megaleledone* from shallow South-American waters prior to the cooling of Antarctica and the associated opening of sea passages between 29–32 million years ago ([25,26], Figure 1). Irrespective of their origin, to become successful members of the Antarctic fauna as they are today, octopods were eventually required to adjust to temperatures as low as −1.9°C.

Survival at such cold temperatures is supported by physiological adjustments that sustain metabolism and motor activity [29,30]. Unlike fishes, which are hypo-osmotic to seawater [31], octopods do not need to fear freezing, as their body fluids are nearly isosmotic to seawater [32] and freeze at about the same temperature of −1.9°C. A major challenge, however, may involve retaining the functionality of the advanced oxygen supply system of coleoid cephalopods. Their closed circulatory system comprises three hearts and contractile veins that pump haemolymph, which is highly enriched with the blue coloured oxygen transport protein haemocyanin (89 mg ml^−1^ in *Megaleledone setebos* [33] or up to >160 mg ml^−1^ in *Loligo vulgaris* [12,34,35]), at blood pressures which are high for invertebrates (e.g. *Enteroctopus dofleini* 5.3-9.3 kPa, [36-38]. Evidence suggests that circulatory support by ventilatory pressure oscillations as well as heart performance may fail at high temperatures and decrease oxygen supply in cephalopods [39,40]. At low temperatures, haemocyanin may cause systemic oxygen shortage due to its decreasing ability to release sufficient oxygen to tissues [33,41].

Low temperatures decrease rate dependent biochemical and metabolic processes [42]. Antarctic ectotherms may compensate for this by fully or partially reversing such effects [43]. To date only few studies have investigated cold compensated features in Antarctic octopods. Garrett and Rosenthal [44] reported accelerated kinetics of potassium channels to enhance nervous signal transduction in the Antarctic octopus *Pareledone sp.*. Daly and Peck [7] observed that Antarctic temperatures lower oxygen consumption rates of *Pareledone charcoti* as predicted from the temperature sensitivity of metabolism of the temperate octopus *Eledone cirrhosa*. Consequently, oxygen consumption rates were considered low and uncompensated in *Pareledone charcoti*. This is even more apparent if one scales oxygen consumption rates to the relatively small size of *Pareledone charcoti*. That is, according to the scaling function of mass-specific oxygen consumption rates for octopods (*M*_O2_ = 3.35 *M*^-0.27^, [45]) an octopus weighing 51 g is expected to consume 1.093 mmol O_2_ kg^−1^ h^−1^ at 0°C (assuming a Q_10_ of 2.12 [46-48]) but *Pareledone charcoti* instead only consumes 0.362 mmol O_2_ kg^−1^ h^−1^ at 0°C [7]. Furthermore, Zielinski et al. [33] studied haemocyanin oxygen binding in the large *Megaleledone setebos* (former *Megaleledone senoi*, [49]) and observed oxygen affinity to be high and irresponsive to temperature, implying poor oxygen unloading and very limited temperature tolerance. However, comparisons of these features with those in octopods from warmer climates are required. It therefore remains unclear whether oxygen supply in Antarctic octopods features adjustments to the cold or simply lacks compensation. It further remains open whether the findings in *Megaleledone setebos* also apply to the much smaller and more common Antarctic octopods of the genus *Pareledone*, and to what extent oxygen supply via haemocyanin differs between the cold water species and octopods that face much higher and more variable temperatures.

Therefore, in this study, we aimed to assess

1. Whether oxygen transport via haemocyanin features modifications that facilitate oxygen supply and thus survival of Antarctic octopods at close to freezing temperatures.
2. Whether oxygen transport properties and related stenothermy reported for *Megaleledone setebos* also occur in other Antarctic octopods.
3. Whether octopods adapted to warmer and broader temperature windows employ diverging strategies to sustain haemocyanin mediated oxygen supply across various temperatures.

To address these objectives, we compared oxygen binding properties, total oxygen carrying capacities as well as protein and ion composition of haemolymph of the abundant Antarctic octopod species *Pareledone charcoti* with two octopod species originating from warmer climates, the South-east Australian *Octopus pallidus* and the Mediterranean *Eledone moschata*.

Here we report specific properties of oxygen transport in the Antarctic octopod *Pareledone charcoti*, which include reduced oxygen affinities and high oxygen carrying capacities, but also a high, thermally sensitive venous reserve that supports eurythermy in *Pareledone charcoti*. We emphasize the general role of haemocyanin in shaping cold tolerance in both cold- and warm-water octopods.

## Results

### Temperature dependent oxygen binding *in vitro*

**Figure 2:**
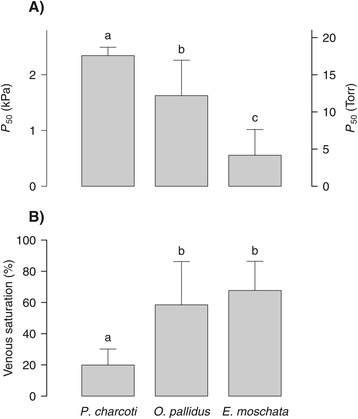
**Lowered affinity of haemocyanin for oxygen in the Antarctic** ***Pareledone charcoti*** **. (A)** Oxygen affinity, expressed as the *P*O_2_ of haemocyanin half-saturation, (*P*_50_), and **(B)** venous oxygen saturation of *Pareledone charcoti* were compared to two octopods originating from warmer waters, *Octopus pallidus* and *Eledone moschata*, at a comparative experimental temperature of 10°C. Calculations refer to an alpha-stat adjusted venous pH of 7.27 at 10°C and a venous *P*O_2_ of 1 kPa. Differing letters indicate significant differences (*P* < 0.05) between species.

*In vitro* changes in oxygen binding by the respiratory pigment haemocyanin were assessed by pH oxygen-saturation analysis (see Methods). At a common temperature of 10°C the haemocyanin of the Antarctic octopod *Pareledone charcoti* displayed a lower affinity for oxygen than haemocyanin of the South-east Australian *Octopus pallidus* and the Mediterranean *Eledone moschata,* reflected in a 1.4- or 4.2-fold higher *P*_50_ (*P*O_2_ at which haemocyanin reaches half-maximum saturation with oxygen (50%)), respectively (Figure 2A, Table 1). Further, at 10°C, the cooperativity of oxygenation dependent proton binding (i.e. expressed as the pH-dependent release of oxygen by haemocyanin and derived from the oxygen carrying capacity and the maximum slope of the pH oxygen-saturation curve, Δmmol O_2_ L^−1^/ ΔpH) was highest in *Pareledone charcoti* (Kruskal Wallis, *χ*^2^(2) = 28.0, *P* < 0.001) compared to *Eledone moschata* (Mann–Whitney, *P* < 0.001) and *Octopus pallidus* (Mann–Whitney, *P* < 0.001, Table 1).

**Table 1:** **Comparison of oxygen binding parameters and cation composition of haemolymph between** ***Pareledone charcoti*** **,** ***Octopus pallidus*** **and** ***Eledone moschata***

**Temperature (°C)**	***P***_50_ **(kPa)**	**Δmmol l** ^−1^ **O**_2_ **/ΔpH**	Bohr coefficient* (pH range 6.7-7.5)	**Δ** ***P***_50_ **(kPa)/°C** ^#^	**Cation concentration (mmol L** ^−1^ **)**				
			**(Δlog** ***P***_50_ **/ΔpH)**		**Mg** ^2+^	**Na** ^+^	**K** ^+^	**NH** ^4+^	**Ca** ^2+^
*Pareledone charcoti*									
0	0.41	1.17							
	(NA, pH 7.42)	(0.88-1.46)							
5	0.7	2.39	−1.22	0.15	52.6	385.5	8.5	6.1	8.8
	(NA, pH 7.34)	(0.47-4.32)	(−1.67 - -0.78)	(NA)	(39.8 – 65.5)	(348.4 – 422.6)	(6.7 – 10.2)	(1.6 – 10.5)	(7.3 – 10.2)
10	2.34	3.17							
	(2.19-2.49, pH 7.27)	(2.51-3.82)							
*Octopus pallidus*									
10	1.63	1.40							
	(0.99-2.26, pH 7.27)	(1.26-1.55)							
15	1.81	2.55	−1.97	0.39	45.6	372.0	10.0	8.6	7.5
	(1.46-2.16, pH 7.19)	(2.24-2.85)	(−2.50 - -1.44)	(0.05 – 0.72)	(41.6 – 49.6)	(356.2 – 387.8)	(7.7 – 12.3)	(3.2 – 13.9)	(6.7 – 8.2)
20	6.07	2.94							
	(3.74-8.40, pH 7.11)	(2.63-3.25)							
*Eledone moschata*									
10	0.56	1.13							
	(0.1-1.02 pH 7.27)	(0.77-1.48)							
15	1.49	1.79	−1.88	0.21	49.9	386.8	8.8	3.0	7.3
	(NA, pH 7.19)	(0.70-2.89)	(−2.22 - -1.55)	(0.11 – 0.30)	(42.2 – 57.6)	(334.0 – 439.5)	(6.2 – 11.4)	(−3.8 – 9.8)	(5.7 – 8.9)
20	2.62	2.08							
	(2.00-3.24, pH 7.11)	(1.79-2.37)							
ANOVA^†^		*F*_2, 149_ = 10.82	*F*_2, 16_ = 3.71	*F*_2, 7_ = 1.58	*F*_2, 17_ = 0.78	*F*_2, 17_ = 0.31	*F*_2, 17_ = 0.85	*F*_2, 17_ = 1.35	*F*_2, 17_ = 2.28
		***P*** **< 0.001**	***P*** **= 0.048**	*P* = 0.271	*P* = 0.475	*P* = 0.739	*P* = 0.446	*P* = 0.285	*P* = 0.133

Numbers in brackets indicate the range of 95% confidence intervals.

*Data at different temperature were pooled for each species.

^#^Based on alpha-stat shifted venous pH and a 10°C temperature interval.

†ANOVA results for between species comparison.

**Figure 3:**
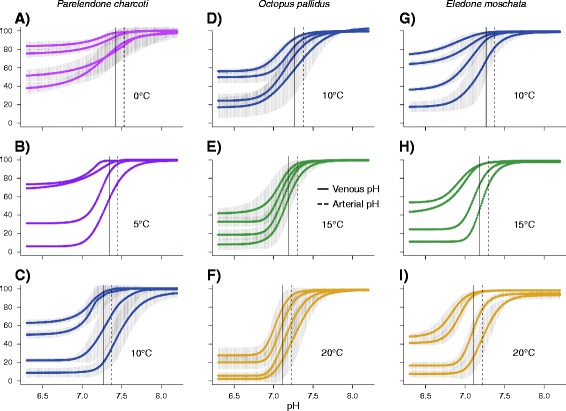
**pH oxygen-saturation curves of haemolymph from Antarctic (A-C), South-east Australian (D-F) and Mediterranean (G-I) octopods.** pH oxygen-saturation curves denote the change of oxygen saturation of haemocyanin from high to low pH at constant *P*O_2_ (21, 13, 4, 1 kPa from left to right) and are most suitable to illustrate the high pH dependence of oxygen binding of cephalopod haemocyanin see [52]. For replicated measurements (*n* = 5–6), means and 95% confidence intervals (shaded area) of fitted pH oxygen-saturation curves are displayed. Replicate measurements could not be performed for *Pareledone charcoti* at 5°C and *Eledone moschata* at 15°C due to insufficient amounts of haemolymph sample. Vertical lines indicate the alpha-stat adjusted arterial (dashed) and venous pH (solid). The ten degree temperature windows cover approximate habitat temperatures for each species.

**Figure 4:**
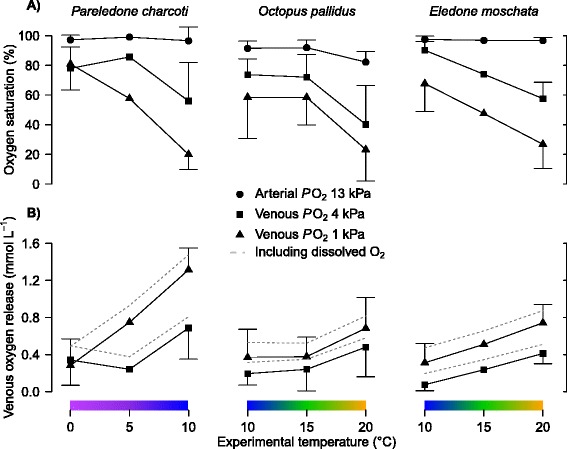
**A) Change of arterial and venous oxygen saturation and B) venous oxygen release by octopod haemocyanin with temperature.** Data refer to an arterial *P*O_2_ of 13 kPa and to venous *P*O_2_ for a resting (4 kPa) and exercised (1 kPa) octopus. Arterial and venous *P*O_2_ were assumed to be constant across temperatures and not determined for the analysed octopod species and instead taken from *Octopus vulgaris* [50,51]. Venous pH values were alpha-stat adjusted for each temperature and arterial pH assumed to be 0.11 pH units higher than venous pH [50]. Venous oxygen release including the contribution by dissolved oxygen is indicated by dashed lines. The ten degree temperature windows cover habitat temperatures for each species except for *Pareledone charcoti*.

Temperature changes affected oxygen binding in all three octopod species, indicated by increased oxygen affinities and diminished cooperativity of oxygenation dependent proton binding towards colder temperatures (Table 1, Figures 3 and 4). In *Pareledone charcoti* and *Eledone moschata*, oxygen affinities increased more steadily, in *Octopus pallidus* however, oxygen affinities remained nearly unchanged between 10-15°C but decreased considerably above 15°C (Table 1, Figure 4). According to the changes in oxygen affinity, oxygen saturation decreased with increasing temperatures (Figure 4A, Note that calculations of oxygen saturation were based on arterial and venous *P*O_2_ and an arterial-venous pH difference determined for *Octopus vulgaris* [50,51] and were assumed to be constant across temperature). However, this drop mostly occurred in the range of low *P*O_2_ between 4 and 1 kPa (Figure 4A). At a *P*O_2_ of 13 kPa, oxygen saturation remained virtually unchanged in *Pareledone charcoti* and *Eledone moschata* and decreased only slightly but significantly by 9.7% in *Octopus pallidus* above 15°C (ANOVA_1-way_, *F*_2, 15_ = 5.40, *P* = 0.017, Figure 4A). pH sensitivity of oxygen affinity expressed as the Bohr coefficient (Δlog *P*_50_/ΔpH) was not significantly affected by experimental temperatures (ANOVA_2-way_, *F*_1, 16_ = 0.36, *P* = 0.555). Among species the lowest Bohr coefficients were found in *Pareledone charcoti* (Table 1). The cooperativity of oxygenation dependent proton binding decreased significantly towards cooler temperatures in all three species (ANOVA_2-way_, *F*_1, 147_ = 114.41, *P* < 0.001, Table 1, Figure 3).

**Figure 5:**
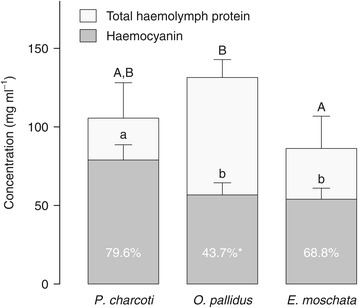
**Total protein and haemocyanin concentrations in haemolymph of cold and warm water octopods.** Haemocyanin concentrations were calculated from the haemolymph oxygen carrying capacity, based on a molecular weight of 3.5 MDa and 70 oxygen binding sites stated for octopod haemocyanin [53]. Total protein concentration was determined according to Bradford [54]. Bars depict means + 95% C.I., *n* = 9–13. Differing letters indicate significant differences (*P* < 0.05) between octopod species for total haemolymph protein (upper case) or haemocyanin concentrations (lower case). White values on bars indicate the fraction of haemocyanin relative to total haemolymph protein and asterisks significant differences between species.

The analysis of inorganic cations in the haemolymph showed no differences between *Pareledone charcoti, Octopus pallidus* and *Eledone moschata* (Table 1). Interestingly, haemocyanin content did not co-vary with or equal total haemolymph protein but differed significantly between species (ANOVA_1-way_, *F*_2, 29_ = 8.98, *p* < 0.001, Figure 5). The highest concentrations of haemocyanin were found in the Antarctic octopod *Pareledone charcoti* (78.9 mg ml^−1^, 95% confidence interval (CI) from 69.2-88.6 mg ml^−1^, Figure 5).

### Implications for blood oxygen transport *in vivo*

In this section, the *in vitro* results are described in terms of their implications for the putative *in vivo* patterns of oxygen binding. At 0°C haemocyanin of *Pareledone charcoti* would release only 16.3% of its bound oxygen assuming an arterial-venous transition from 13 to 1 kPa *P*O_2_ and pH 7.53-7.42 (Figure 3A, Figure 4). Even at low pH (<6.4) and low oxygen tensions (1kPa *P*O_2_), 33.6% (28.4-38.8) of the oxygen would remain bound to the Antarctic haemocyanin. For comparison, within the range of their habitat temperature from 10 to 20°C, haemocyanins of *Octopus pallidus* and *Eledone moschata* would release between 33.0-60.0% and 29.8-70.0% oxygen, respectively (Figure 3D-I, Figure 4).

**Figure 6:**
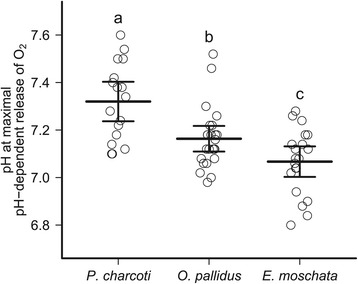
**pH at which the pH-dependent release of oxygen by haemocyanin becomes maximal.** Comparison between the Antarctic *Pareledone charcoti*, the South-east Australian Octopus *pallidus* and the Mediterranean *Eledone moschata* at an experimental temperature of 10°C. Calculations include pH oxygen-saturation curves from all analysed *P*O_2_. Letters indicate significant differences (*P* < 0.05) between species. Data from different *P*O_2_ were pooled due to similar effects by *P*O_2_ among species.

Haemocyanin of *Pareledone charcoti* showed the lowest venous oxygen saturation at a common temperature of 10°C, at a venous *P*O_2_ of 1 kPa and a venous pH of 7.27 (Figure 2B). At 10°C the Antarctic haemocyanin thus has the potential to release far more oxygen (on average 76.7%, 95% CI 68.6% to 84.8%) upon each cycle than the warm-water octopods *Octopus pallidus* (33.0%, 5.0-60.9) and *Eledone moschata* (29.8%, 9.9-49.7, Figure 3C, D, G, Figure 4A). This is mostly due to an increased pH dependent release of oxygen by haemocyanin in *Pareledone charcoti* (Figure 3C, D, G), with maxima occurring 0.16 or 0.25 pH values above those of *Octopus pallidus* or *Eledone moschata* respectively (Figure 6).

Surprisingly, the Antarctic *Pareledone charcoti* has a larger capacity to carry oxygen in its haemolymph than *Octopus pallidus* or *Eledone moschata* (ANOVA_1-way_, *F*_2, 31_ = 12.57, *p* < 0.001, Table 2), due to the highest haemocyanin content of all three species (Figure 5). This increased capacity for oxygen transport in *Pareledone charcoti* is further enhanced by high levels of dissolved oxygen at 0°C (359.5 μmol L^−1^, 35 psu (practical salinity units, Figure 4B) accounting for 18.5% of the total haemolymph oxygen content and up to 42% of the oxygen released to the tissue in *Pareledone charcoti* (assuming an arterial-venous transition from 13–1 kPa *P*O_2_ and pH 7.53-7.42). The contribution of dissolved oxygen is also significant in the warm-water octopods *Octopus pallidus* and *Eledone moschata*, within the range of their habitat temperatures between 10-20°C, amounting to between 17-20% or 18-21%, respectively, of total haemolymph oxygen content and 30-16% or 34-15%, respectively, of the oxygen eventually released to tissues (assuming an arterial-venous transition from 13–1 kPa *P*O_2_, Figure 4B).

**Table 2:** **Comparison of oxygen carrying capacities**

**Species**	**Oxygen carrying capacity (mmol L** ^−1^ **)**	**Source**
*Megaleledone setebos*	1.86	[33]
*Octopus macropus*	1.60	[55]
*Pareledone charcoti*	1.58 (1.38-1.77, 14)	This study
*Enteroctopus dofleini*	1.36	[56]
*Octopus pallidus*	1.13 (0.98-1.29, 10)	This study
*Eledone moschata*	1.08 (0.94-1.22, 10)	This study
*Octopus vulgaris*	0.61	[12]
Red blooded Antarctic fishes	1.77 (1.44-2.09, 11)	[8]

Values are listed in descending order. Numbers in brackets indicate 95% confidence intervals and samples size *n*, when available. Oxygen carrying capacities of red blooded Antarctic fishes were calculated from their haemoglobin content, based on a molecular weight of 66 kDa [57], and averaged for 11 species.

## Discussion

Comparing the haemocyanins of the Antarctic octopod *Pareledone charcoti* with those of the warmer-water octopods *Octopus pallidus* and *Eledone moschata* reveals differences and properties of the respiratory pigment that assist oxygen supply at close to freezing temperatures but also support an extended range of oxygen dependent thermal tolerance in the Antarctic species. Haemocyanin functional properties in *Eledone moschata* constrain oxygen supply by haemocyanin at its lower temperature margin of 10°C. In *Octopus pallidus*, however, oxygen affinities decrease strongly above 15°C but stabilise at 10°C, suggesting a dual strategy to improve oxygen supply at both its upper and lower temperature margins.

### Blood oxygen transport in the cold

Due to the exothermic binding of oxygen in cephalopod haemocyanins, oxygen affinity increases towards colder temperatures and may severely hamper oxygen release to tissues at the sub-zero temperatures [58] prevailing in the Antarctic Ocean. Our results show that *Pareledone charcoti* attenuates this detrimental effect by means of lowered oxygen affinity of the haemocyanin (Figure 2A, Table 1). Such lowered, cold-compensated oxygen affinities are not unique to *Pareledone charcoti* and the respiratory pigment haemocyanin, but were also observed in red-blooded Antarctic fishes such as *Dissostichus mawsoni* (*P*_50_ of 1.93 kPa at pH 8.16 and −1.9°C, [17]) or *Pagothenia borchgrevinki* (2.8 kPa at pH 8.1 and −1.5°C), whose oxygen affinities were much lower than those of temperate fish extrapolated to the same temperatures [59].

**Figure 7:**
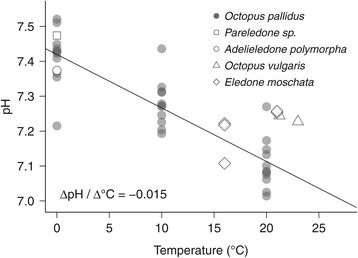
**Observed alpha-stat pH pattern for octopus haemolymph.** The temperature dependent change of pH was determined for thawed *Octopus pallidus* haemolymph at 0°C, 10°C, and 20°C. Venous pH of the other species refer to freshly sampled and analysed haemolymph. pH were corrected to the free hydrogen ion scale by subtracting an experimentally determined offset of −0.136 (0.130-0.142, *n* = 87) pH units to account for the high ionic strength of cephalopod haemolymph [61]. Sources: *Octopus pallidus*, *Pareledone sp.*, *Adelieledone polymorpha* (Strobel and Oellermann 2011, unpublished); *Eledone moschata* (Strobel and Mark 2010, unpublished); *Octopus vulgaris* [50,51].

Allosteric effectors (e.g. ATP) may strongly contribute to decreased oxygen affinities of the haemoglobins of Antarctic fishes [17]. *Pareledone charcoti* however, relies on modifying the intrinsic properties and the pH sensitivity of its haemocyanin. The only known allosteric effectors in octopod haemolymph, inorganic ions, particularly magnesium [60,61], are not regulated and found at levels similar to those in sea water (54.2 mmol L^−1^ at 35 psu, [62]) and similar to those in haemolymph of other octopods (i.e. *Octopus pallidus*, *Eledone moschata*, Table 1; *Eledone cirrhosa*, 54.6 mmol L^−1^ [32]). This confirms that cephalopods do not regulate haemolymph magnesium concentrations to modulate oxygen binding. Instead, *Pareledone charcoti* increases oxygen release via a higher pH dependent release of oxygen by haemocyanin and by a pH sensitive range of oxygen binding located at higher pH values than seen in the warm water octopods (Figure 3C, D, G, Figure 6), which aligns with the cold-induced alpha-stat shift of venous pH (Figure 7).

### Compensation for incomplete oxygen release

Although *Pareledone charcoti* has experienced a decrease in oxygen affinity of its haemocyanin oxygen release is far from being complete at 0°C as more than 77% of the oxygen remains bound to haemocyanin (assuming an arterial-venous transition from 13 to 1 kPa *P*O_2_ and pH 7.53-7.42, Figures 3A and 4A). The major factors contributing to the projected incomplete oxygen unloading at 0°C are the i) cold-induced increase of affinity of haemocyanin for oxygen, ii) reduced pH dependent release of oxygen by haemocyanin and iii) alpha-stat shift of haemolymph pH towards higher pH (Figures 6 and 7). In fact, increased oxygen affinity and reduced release of oxygen at colder temperatures are consistently reported for octopods ([12,13] for review) and assumed to be due to a more rigid structure of the haemocyanin molecule [61]. The alpha-stat pattern of haemolymph pH changes observed for octopods (Figure 7) has also been reported for squids [63], suggesting that temperature dependent changes of haemolymph pH affect oxygen supply in most if not all cephalopods. Melzner et al. [39] illustrated that the interplay of these factors lead to a venous oxygen release of less than 10% in *Sepia officinalis* at 10°C and 1.7 kPa *P*O_2_, and accordingly, to only ~22% or ~5% oxygen release at 0°C and 1.0 or 1.7 kPa *P*O_2_, respectively, in the Antarctic octopod *Megaleledone setebos* [33]. Although *P*O_2_ values below 1 kPa may further improve oxygen unloading, it is questionable whether the remaining oxygen gradient to mitochondria would be steep enough to maintain oxygen flux [39]. Therefore, poor oxygen unloading in *Pareledone charcoti* at 0°C due to high oxygen affinity, lowered pH dependent release of oxygen by haemocyanin and high venous pH are well in line with previous notions describing these factors to be crucial in defining limits of oxygen supply in the cold [13,39,58].

Most surprisingly, *Pareledone charcoti* compensates for poor oxygen unloading by considerably increasing haemocyanin concentrations. It thereby carries 40% or 46% more haemocyanin-bound oxygen in its haemolymph than *Octopus pallidus* or *Eledone moschata*, respectively (Figure 5). Overall, oxygen carrying capacities of the Antarctic octopods *Pareledone charcoti* and *Megaleledone setebos* rank among the highest reported for octopods and resemble those of red-blooded Antarctic fishes (Table 2). This and the presence of deeply blue-colored haemolymph in many other Antarctic octopods (*Adelieledone polymorpha, Pareledone spp., Benthoctopus* sp., M. Oellermann, pers. obs.) not only underlines the dependence of Antarctic octopods on high haemocyanin concentrations but also contrasts the general finding of reduced erythrocyte and blood pigment concentrations in red-blooded Antarctic fishes [8] or Antarctic crustaceans [64]. It appears that red-blooded Antarctic fishes depend less on their oxygen transport protein than Antarctic octopods, despite higher rates of oxygen consumption (e.g. *Trematomus hansoni* 22.4 mg O_2_ kg^−1^ (wet mass) h^−1^ [65] vs. *Pareledone charcoti* 10.2 mg O_2_ kg^−1^ (wet mass) h^−1^ [7]). This may reflect a lower degree of capillarisation in the cephalopods [66] or the lower oxygen binding capacity of haemocyanin compared to fish haemoglobin [67]. However, we can presently not exclude that high haemocyanin protein concentrations serve other cold compensated processes as well.

The reduction of haemoglobin content in red-blooded Antarctic fishes has been interpreted to balance the increase in blood viscosity at low temperatures [68,69]. One therefore wonders why Antarctic octopods evolved to maximize the concentration of an extracellular protein, which increases viscosity even further? This may be best explained by either one or all of the following reasons, i) an increase in the fraction of haemocyanin in extracellular protein without causing higher levels of haemolymph proteins (Figure 5), ii) the non-existence of anti-freeze proteins that can largely contribute to blood protein levels in Antarctic fishes (e.g. 32 mg ml^−1^ or ~35% of total blood protein concentration in *Dissostichus mawsoni*, [8,31]) and increase blood viscosity [70] and iii) haemocyanin concentrations below viscosity limits. Squids living in temperate and subtropical waters were reported to have haemocyanin in excess of 160 mg ml^−1^ (*Loligo vulgaris* and *Loligo pealei*, [12,61,71]), whereas maximum haemocyanin levels of *Pareledone charcoti* seen in the present study were 106.8 mg ml^−1^. However, maximum tolerated haemocyanin levels may be far lower at 0°C due to increasing blood viscosity towards colder temperature [68]. We conclude that as a trade-off, increased haemocyanin concentrations occur at the expense of elevated viscosity. The ability to maximize haemocyanin levels at sub-zero temperatures supports *Pareledone charcoti* in compensating for the poor oxygen unloading by its haemocyanin.

Oxygen supply is further enhanced by high levels of physically dissolved oxygen, as oxygen solubility increases with decreasing temperatures (e.g. by 40% from 15°C to 0°C, [72]). Consequently, dissolved oxygen contributes 18.5% to total haemolymph oxygen content. Given the small degree of putative venous oxygen unloading in *Pareledone charcoti* (below 20%), even at very low *P*O_2_ (1 kPa), physically dissolved oxygen contributes a large fraction (42%, assuming an arterial-venous transition from 13 to 1 kPa *P*O_2_ and pH 7.53-7.42, Figure 4B) of the oxygen supplied to tissues. Red-blooded Antarctic fishes also benefit from high ambient oxygen levels in the cold [73] and combined with low metabolic rates [5,65], this may be the key to the reduction in haemoglobin levels [74]. For *Pareledone charcoti* it rather seemed inevitable to increase haemocyanin concentrations, despite high dissolved oxygen levels, reduced oxygen affinity and metabolic rates lower than in fish [7]. Sustaining high haemocyanin levels may be energetically costly but may alleviate the pressure to evolve functional changes enabling complete oxygen unloading at 0°C. Such ‘complete’ compensation may not be possible considering the enormous size (3.5 MDa) and multimeric complexity of the haemocyanin molecule [53]. Although *Octopus pallidus* and *Eledone moschata* live at higher temperatures and lower dissolved oxygen levels, dissolved oxygen still contributes significantly to oxygen transport, especially towards colder temperatures when their haemocyanin increasingly fails to supply oxygen to tissues (Figure 4B).

### Temperature sensitivity of oxygen transport

The increase in oxygen affinity and decrease of pH dependent oxygen release towards colder temperatures (Table 1, Figure 3) results in progressively reduced capacities to unload oxygen in all three octopod species. The change of oxygen affinity with temperature in *Pareledone charcoti* and *Eledone moschata* (Table 1) conforms with findings in other octopod species (in Δ*P*_50_ (kPa)/°C: 0.24, *Enteroctopus dofleini*; 0.20, *Octopus vulgaris* [12]; 0.10, *Eledone cirrhosa*; 0.14, *Octopus vulgaris* [75]). The temperature dependence of these data from the literature would have been even more pronounced if *P*_50_ were determined at an alpha-stat adjusted pH and not at a fixed pH of 7.4 across temperatures. Consequently, oxygen release and uptake by haemocyanin strongly depend on temperature in numerous octopod species, which poses a considerable challenge in the cold where high oxygen affinities diminish oxygen release to tissues [12,39].

However, some species deviate from this pattern, such as the Antarctic octopod *Megaleledone setebos*. The response of its haemocyanin oxygen affinity to temperature changes (0.01 kPa ΔP_50_/°C, [33]), was 8–32 times less than that in any other octopod studied and 12 times less than the respective change in *Pareledone charcoti*. Despite similarities in oxygen affinity and oxygen carrying capacity between these two Antarctic octopods, this difference is striking. Thus, in addition to enhanced oxygen carrying capacities, two alternating strategies emerge to compensate for excessively high oxygen affinities in the cold: 1) A general decrease in oxygen affinity at all temperatures but with high sensitivity to temperature maintained as in *Pareledone charcoti* or 2) a considerable decrease of temperature sensitivity leading to reduced oxygen affinity at low temperatures only, as in *Megaleledone setebos*. Interestingly, *Octopus pallidus* seems to take advantage of both strategies as oxygen affinity barely changes between 10 and 15°C but strongly decreases between 15 and 20°C (Table 1, Figure 4). As a consequence, oxygen supply is sustained at temperatures below 10°C but also improves rapidly at higher temperatures (>15°C) when metabolic demand for oxygen increases. *Eledone moschata*, on the other hand, faces a constant increase of oxygen affinity and thus insufficient oxygen supply below 10°C (Table 1, Figure 4), which would contribute to cold-death at around 6°C (F. C. Mark, pers. obs.). Thus with respect to haemocyanin-mediated oxygen supply, *Octopus pallidus* seems to tolerate cold temperatures better than *Eledone moschata*.

Within the studied temperature ranges, warming hardly compromises the capacity for oxygen loading at the gills but does compromise oxygen release to tissues in all three octopods (Figure 4A). Only *Octopus pallidus* will experience reduced arterial oxygen loading above 15°C (assuming arterial *P*O_2_ is 13kPa), which however is paralleled by increased venous oxygen unloading (Figure 4). This conforms with findings in other cephalopods and indicates that temperature changes affect venous unloading more than arterial oxygen loading (*Megaleledone setebos, Sepia officinalis*, [33], *Dosidicus gigas*, [76], *Todarodes sagittatus*, [12]). Only few species like the giant squid *Architeuthis monachus* experience significantly reduced arterial saturation at higher temperatures [77]. Thus, in octopods, oxygen loading at the gills seems largely safeguarded at habitat temperatures and normoxic conditions and may only be compromised at low ambient oxygen levels.

The pH sensitivity of oxygen binding expressed as the Bohr coefficient remained unaffected by temperature changes in all three octopods, unlike in *Megaleledone setebos* or *Sepia officinalis*, whose Bohr coefficients decreased with falling temperatures [33]. Octopods may benefit from low Bohr coefficients in the cold, equivalent to a switch from pH dependent to *P*O_2_ dependent oxygen release. This may preserve the venous oxygen reserve when metabolic rate is low and largely covered by elevated physically dissolved oxygen levels. The lower Bohr coefficient of *Pareledone charcoti* may also reflect its low activity mode of life in cold Antarctic waters where rapid pH dependent mobilization of the venous reserve is not required. Conversely, the strong increase in the Bohr coefficient of *Megaleledone setebos* haemocyanin during warming to 10°C (−2.33) challenges effective oxygen release outside of the animal’s usual thermal range [33]. In contrast to the findings in the Antarctic species, the maintenance of high Bohr coefficients in cold exposed temperate *Octopus pallidus* and subtropical *Eledone moschata* may reflect suboptimal or even impaired oxygen supply at their lower temperature margins.

### Haemocyanin supports eurythermy

**Figure 8:**
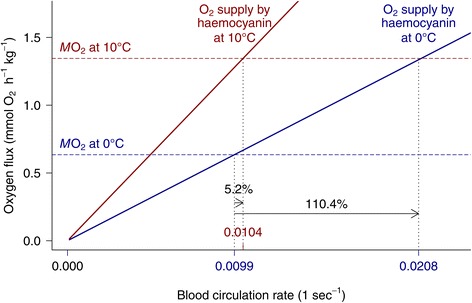
**Additional release of oxygen by haemocyanin relieves the circulation system of** ***Pareledone charcoti*** **at 10°C.** Oxygen that remained bound to haemocyanin at 0°C (blue) was largely liberated at 10°C (red), and thereby reduces the need for increased blood circulation (i.e. expressed as number of times to circulate the whole blood volume per second, 5.2% vs. 110.4% increase in circulation) to match an increased oxygen demand at 10°C. Oxygen supply rates (O_2_ release from haemocyanin between 13 and 4 kPa *P*O_2_, solid lines) match oxygen consumption rates of *Pareledone charcoti* (mean *M*O_2_ ± SD, 0.63 mmol O_2_ kg^−1^ (wet mass) h^−1^ ± 0.12, at 0°C, vertical dashed lines, taken from [7]) at the intersections of both rates at 0°C or 10°C (values indicated on x axis). Oxygen supply comprises the oxygen transported by haemocyanin only without contributions by dissolved oxygen or oxygen absorbed via the skin. The *M*O_2_ at 10°C was interpolated assuming a Q_10_ of 2.12 (average Q_10_ for Octopoda taken from [46-48]. The blood volume was assumed to be 5.2% (v/w) based on average literature values from *Octopus vulgaris* and *Enteroctopus dofleini* [78,79].

*Pareledone charcoti* benefits from its thermally sensitive oxygen binding during warming, as much of the bound oxygen is liberated then (Figure 4A). Figure 8 models the relationship of oxygen supply by haemocyanin, oxygen consumption and blood circulation rate at 0°C and 10°C and an assumed haemolymph volume of 5.2% (v/w, based on average literature values from *Octopus vulgaris* and *Enteroctopus dofleini* [78,79]. If oxygen supply by haemocyanin would remain constant from 0°C to 10°C (i.e. 0.34 mmol O_2_ L^−1^ at an arterial-venous transition from 13 to 4 kPa *P*O_2_ and pH 7.53-7.42, Figure 4B), blood circulation would need to increase by 110.4% to match a rise of oxygen consumption from 0.63 mmol O_2_ kg^−1^ (wet mass) h^−1^ at 0°C (taken from [7] to 1.35 mmol O_2_ kg^−1^ (wet mass) h^−1^ at 10°C (*M*O_2_ was extrapolated to 10°C using an average Q_10_ of 2.12, taken from [46-48]. However, due to the large increase of oxygen supply by haemocyanin at 10°C (Figure 4B), demand for oxygen requires only a minimal increase in circulatory performance by 5.2% in *Pareledone charcoti* (Figure 8). Consequently, haemocyanin in *Pareledone charcoti* plays a major role in buffering oxygen demand when temperature increases and drastically reduces the workload for other circulatory components, particularly the hearts, which often limit ectotherm performance at high temperatures [80] such as in the cephalopod *Sepia officinalis* [40], fishes [81,82], or crustaceans [83,84]. Hence, haemocyanin function extends the range of oxygen dependent warm tolerance of *Pareledone charcoti*, which may cope far better with higher temperatures than *Megaleledone setebos,* whose haemocyanin, due to its low temperature sensitivity and extreme Bohr coefficient, barely supports oxygen supply at higher temperatures [33]. In fact, *Pareledone charcoti* sustains fully aerobic metabolism up to 8-10°C and thus tolerates elevated temperatures well [85]. Although both species are closely related and likely originate from shallow Southern Ocean waters (i.e. possess an ink sac [28], Figure 1), this may in part reflect the different geographic and vertical distribution of the two species. *Megaleledone setebos* is a circum-Antarctic species found between 30–850 m and most frequently below 100 m [49] where temperatures remain close to freezing all year round [86]. *Pareledone charcoti* inhabits the waters around the Northern Antarctic Peninsula mostly from less than 120 m [87] to very shallow waters (intertidal < 3 m, F. C. Mark, pers. obs.) and even visits tidal water pools [88] where water temperatures vary (e.g. from −0.5°C to 10.7°C during summer [86,89]). Our data provide first evidence that haemocyanin supports oxygen dependent eurythermy in an Antarctic invertebrate ectotherm and conform to analogous findings in the temperate, eurythermic crab *Carcinus maenas* [90]. Considering the strong warming trend at the Antarctic Peninsula [91], *Pareledone charcoti* may eventually benefit from its capacity to adjust oxygen supply to more variable temperatures than more stenothermal species.

## Conclusions

This study highlights the importance of the oxygen transport pigment haemocyanin in octopods with regard to temperature compensation. In comparison to findings in the south east Australian *Octopus pallidus* and the Mediterranean *Eledone moschata*, the analysis of blood oxygen binding in the Antarctic octopod *Pareledone charcoti* revealed properties of its blood pigment haemocyanin that support oxygen supply in the cold but at the same time maintain haemocyanin function in the warmth. Significantly lower oxygen affinity but incomplete deoxygenation of haemocyanin in *Pareledone charcoti* resulted in sustained but poor oxygen unloading at 0°C, which however, was compensated for by high levels of dissolved oxygen as well as elevated haemocyanin concentrations and thus oxygen carrying capacities. In contrast to the stenothermic Antarctic octopod *Megaleledone setebos*, *Pareledone charcoti* benefits from a thermally sensitive haemocyanin that extends oxygen supply at warmer temperatures and thus supports oxygen dependent eurythermy. Compromised oxygen release from haemocyanin in the cold underlines the crucial role of the pigment for defining cold tolerance not only in Antarctic but also in warmer water octopods. While some warmer water octopods succeed to extend oxygen supply at cold temperature margins by e.g. reduced temperature sensitivity of oxygen binding in the cold others fail to do so. However, for a complete picture of thermal tolerance in *Pareledone charcoti* and the other octopods much more information is needed regarding *in vivo* haemolymph *P*O_2_ and pH under rest and exercise, the role of cardiac and circulatory performance, aerobic scope and growth rates across various temperatures as well as acclimation capacities. Only then may one predict the future role of this abundant group of ectotherms in a rapidly warming ecosystem.

## Methods

### Study design

To assess whether blood oxygen transport in Antarctic octopods exhibits features that support oxygen supply in the cold, we compared oxygen binding properties, total oxygen carrying capacities as well as protein and ion composition of haemolymph of the Antarctic octopod *Pareledone charcoti* with two octopods originating from warmer waters - *Octopus pallidus* and *Eledone moschata*. Comparisons were performed at habitat temperatures and at a common temperature of 10°C, assuming that all haemocyanin types remained functional at these temperatures. To evaluate if earlier observations for *Megaleledone setebos* haemocyanin apply to other Antarctic octopods as well, we chose *Pareledone charcoti* as a representative of the most abundant and more typically sized genus *Pareledone* [20,87]. Temperature sensitivity of oxygen binding was analysed in all three species to assess the role of octopod haemocyanin in oxygen supply across the respective habitat temperatures.

### Animals and sampling

The octopod *Pareledone charcoti* belongs to the Southern Ocean endemic octopod family Megaleledonidae (Figure 1, [28]) and inhabits the shallow shelf area around the Antarctic Peninsula [87] with temperatures varying between −1.9 to +2°C [92]. Using bottom trawls, specimens were collected on the RV Polarstern cruise ANTXXVIII/4 in March 2012, at depths between 90–470 m around Elephant Island (61°S, 56°W, cruise details [93]), where temperatures ranged between 0.1 to 1.6°C and salinities between 34.3-34.6 psu. *Octopus pallidus* belongs to the non-polar shallow water octopod family Octopodidae (Figure 1, [28]) and inhabits the well mixed waters in South East Australia with habitat temperatures ranging from 12-18°C from winter to summer [94,95]. Specimens were caught in July 2012, between 40–50 m depth, in the western Bass Strait near Stanley (41°S, 145°E) by fishermen (T.O.P. Fish Pty Ltd.) using plastic octopus pots and then transported and kept overnight in large tanks connected to a flow-through seawater system at the Institute for Marine and Antarctic Studies, Hobart. *Eledone moschata* belongs to the non-polar octopod family Eledonidae (Figure 1, [28]) and occurs all over the Mediterranean Sea mainly at depths between 0–200 m [96,97]. Specimens were fished in November 2008, between 20–40 m depth using bottom trawls, in the northern Adriatic Sea near Chioggia, where habitat temperatures vary largely, both by depth and seasonally, between approximately 10-23°C [98]. Average body masses were 32.5 g (28.0-37.0) for *Pareledone charcoti*, 563.3 g (481.0-645.7) for *Octopus pallidus* and 52.9 g (35.6-70.2) for *Eledone moschata*. All animals were anaesthetised in 3% ethanol [99] until non responsive, then ventrally opened to withdraw haemolymph from the cephalic vein, the afferent branchial vessels and the systemic heart and finally killed by a final cut through the brain (Animal research permit no. 522-27-11/02-00 (93), Freie Hansestadt Bremen, Germany and animal ethics approval no. AEC12-43, La Trobe University, Bundoora, Australia). Haemolymph samples were spun down at 15.000 *g* for 15 min at 0°C to pellet cell debris and supernatants were stored at −20°C.

### Phylogenetic analysis

To illustrate phylogenetic relationships of *Pareledone charcoti*, *Octopus pallidus* and *Eledone moschata*, we performed Bayesian phylogenetic analysis using five genes, the mitochondrial genes cytochrome oxidase subunit I and III and the nuclear genes rhodopsin, octopine dehydrogenase and 16S rDNA of 16 octopodiform species (GenBank accession numbers in Additional file 1). Sequences for each gene were aligned separately using the MUSCLE plugin of Geneious 7.1.7 [100]. The resulting alignments were curated using GBlocks 0.91b [101,102] tolerating gap positions within final blocks and concatenated to one data set. Based on the Akaike Information Criteria [103], JModeltest 2.1.5 [104], identified the GTR + I + G model as the best substitution models for the concatenated data set. Bayesian trees were constructed using MrBayes (v. 2.0.3) [105] as implemented in Geneious running at least two independent Monte Carlo Markov Chain (MCMC) analysis with 2,000,000 generations sampled every 2,000 generations. The appropriate burnin was chosen based on the resulting traces, which showed a stationary distribution before 10% of the MCMC chain. *Vampyroteuthis infernalis* and *Argonauta nodosa* were used as outgroups.

### Blood characteristics

#### Oxygen binding properties

Oxygen binding of octopod haemocyanin was characterised using a modified diffusion chamber (for details see [106]), which simultaneously measures pigment oxygenation and pH in a 15 μl sample. Experiments were performed at common habitat temperatures of each species (0°C *Pareledone charcoti*, ~10-20°C *Octopus pallidus*, ~10-20°C *Eledone moschata*) and at a comparative temperature of 10°C. The temperature was monitored and controlled via a temperature sensor (PreSens, Germany) and a connected water bath with a thermostat (LAUDA Ecoline Staredition RE 104, Germany), filled with an anti-freeze solution (20% ethylene glycol, AppliChem, Germany). Prior to measurements, aliquots of 18 μl thawed haemolymph were spun down to collect all liquid at the bottom of a 1.5 ml microcentrifuge tube (5 sec at 1000 *g*), preconditioned with pure oxygen gas to deplete dissolved carbon dioxide (CO_2_) and 0.6-0.9 μl of 0.2 mmol L^−1^ NaOH (8–12 μmol L^−1^ final concentration) added to raise haemolymph pH above 8.0 to ensure full oxygenation. To account for the pronounced pH sensitivity of cephalopod pigments [52], changes of pH and absorbance were recorded at 347 nm in 15 μl haemolymph, at continuously decreasing *P*CO_2_/pH (0–10 kPa/~ pH 8.1-6.8) and four constant *P*O_2_ levels (21, 13, 4, 1 kPa, after Pörtner [52]), with gas mixtures being supplied by gas mixing pumps (Wösthoff, Germany). The spectrophotometer (USB2000+, Ocean Optics, USA) was set to 15 milliseconds integration time, 100 scans to average and 30 seconds measurement intervals and calibrated by recording light and dark spectra without sample. Prior to each experiment, the pH optode was calibrated in MOPS-buffered (40 mmol L^−1^, 3-(N-Morpholino) propanesulfonic acid), filtered artificial seawater (35 psu) equilibrated to the respective experimental temperature at six pHs ranging from 6.7 to 8.1. The pH of buffers was checked with a pH glass electrode (InLab Routine Pt1100, Mettler Toledo, Germany) and a pH meter (pH 330i, WTW, Germany), calibrated with low ionic strength NIST pH standards (AppliChem, Germany, DIN19266) and corrected to Free Scale pH with Tris-buffered seawater standard (Dickson, CO2 QCLab, batch 4 2010, USA, [107]) equilibrated at the same temperature. The pH signal was corrected for instrumental drift and for effects of auto-fluorescence intrinsic to haemolymph [106] and is presented here on the free hydrogen ion scale [108].

Each experiment involved the calibration with pure oxygen or nitrogen to obtain maximum and minimum oxygenation signals. Correct pigment saturation was calculated by continuous readjustments of the maximum oxygenation signal to account for its linear drift observed during the course of an experiment [106,109]. While the maximum oxygenation signal did not change within the range of temperatures employed for each species, the minimum oxygenation signal increased towards colder temperatures due to incomplete oxygen unloading, even under pure nitrogen and low pH (<6.6). For such experiments we predicted minimum absorbance from a reference wavelength of the first recorded spectrum with an uncertainty of 5%, based on a linear regression model applied to 20 experiments with fully deoxygenated pigments (Additional file 2).

To determine the total oxygen bound to octopod haemocyanin (i.e. oxygen carrying capacity) 10 μl of thawed haemolymph were equilibrated with pure oxygen gas in a microcentrifuge tube on ice for 10 min and transferred with a gas tight Hamilton syringe to a gas sealed chamber containing 2 ml of a 32°C warm cyanide solution (55 mmol L^−1^ potassium cyanide, 3 g L^−1^ [110]). Two high-resolution Oxygraph-2 k respirometers (OROBOROS Instruments, Innsbruck, Austria) and DatLab analysis software (version 5.1.0.20) recorded the liberated oxygen (nmol ml^−1^), corrected for air pressure, temperature and background oxygen flux. For each experiment, the respirometers were calibrated with air at the beginning and sodium dithionite added at the end for a zero calibration. The contribution of dissolved oxygen was experimentally determined by the addition of ice-cold, oxygen saturated, filtered seawater (35 psu). The observed change of oxygen concentration was then subtracted from the haemolymph measurements to obtain the final oxygen carrying capacity of haemocyanin.

#### Alpha-stat pattern of haemolymph pH

To be able to analyse oxygen binding parameters at various temperatures, we assessed whether the pH of octopod haemolymph follows an alpha-stat pattern [111] or remains constant across temperatures (i.e. pH stat pattern). Replicated measurements on 20 μl thawed haemolymph of *Octopus pallidus* at 0°C, 10°C and 20°C, using a micro pH electrode (InLab Ultra-Micro, Mettler Toledo, Germany), showed that pH decreases linearly with temperature (*b* = −0.0153 pH units / °C, *t*_31_ = −9.71, *P* < 0.001, *R*^2^ = 0.75, Figure 7), analogous to an imidazole buffered system (−0.0162 pH units / °C, [111]). pH analysis of freshly sampled haemolymph from other species confirmed that octopod haemolymph follows this linear pH-temperature relationship *in vivo* (Figure 7) and therefore exhibits an alpha-stat pattern as also demonstrated for squid [63]. Hence, venous and arterial pH were determined on this basis for various temperatures.

#### Protein and ion concentration

Protein content of octopod haemolymph was determined according to Bradford [54]. Thawed haemolymph was diluted tenfold (v:v) with stabilising buffer (in mmol l^−1^, 50 Tris–HCl, 5 CaCl_2_ 6 H_2_O, 5 MgCl_2_ 6 H_2_O, 150 NaCl, pH 7.47 at 22°C) and 5 μl mixed with 250 μl Bradford reagent (Bio-Rad, Germany). Following 10 min incubation at room temperature, absorbance was recorded at 595 nm using a microplate spectrophotometer (PowerWave HT, BioTek, U.S.A.). Bovine albumin serum served as protein standard to calculate total protein concentrations.

Concentrations of functional haemocyanin (c(Hc)) in haemolymph were derived from the oxygen carrying capacity $\left({C}_{O_2}\right)$, the molecular weight (MW) of octopod haemocyanin (3.5 MDa) and its 70 oxygen binding sites *n*(*HcO*_2_), [53], Equation 1).

1 $$ c(Hc)=\frac{C_{O_2}}{n\left(Hc{O}_2\right)}MW $$

Results from tests with thawed haemolymph of *Octopus vulgaris* (mean ± S.D., 54.3 ± 6.9 g L^−1^) agreed well with data obtained from freshly observed haemolymph via atomic absorption spectroscopy (55.9 ± 7.4 g L^−1^, [35]), which not only confirmed the accuracy of our approach but also that storage at −20°C does not affect the oxygen binding capacity of cephalopod haemolymph [61].

Although inorganic ions such as Mg^2+^ or Na^+^ can affect oxygen affinity in octopods [60], they seem to be insignificant regulators of oxygen binding in most cephalopods [61]. To verify this for the observed species, we diluted haemolymph 400-fold with deionised water and determined cation concentrations by ion chromatography (ICS-2000, Dionex, Germany) following cation separation by an IonPac CS 16 column (Dionex, Germany) with methane sulfonic acid (MSA, 30 mmol L^−1^) as an eluent at 0.36 ml min^−1^ flow rate and 40°C. Ion concentrations were derived from the peaks corresponding to the Dionex Combined Six Cation Standard-II.

### Data analysis

Processing of raw data and statistical analysis was performed using the ‘R’ statistical language R Core [112]. Recordings of pH and pigment oxygenation were time-matched and analysed in pH/saturation diagrams, most suitable for pH sensitive pigments like cephalopod haemocyanin [52]. An empirical five parameter logistic model was applied (‘drc’ add-on package, [113]) to fit sigmoidal curves to the pH/saturation data [106]. Resulting pH oxygen-saturation curves display the change of pigment oxygenation with pH at constant *P*O_2_. Affinity of haemocyanin to oxygen, expressed as *P*_50_, denotes the log_10_ of the *P*O_2_ corresponding to a pH oxygen-saturation curve and the intersecting pH at half saturation (pH_50_, [52]). Δlog_10_*P*_50_ was then plotted versus ΔpH_50_ to obtain the Bohr coefficient from the resulting linear regression slope. Cooperativity of oxygenation linked proton binding was expressed as the change of molar oxygen concentration per pH unit (Δmmol O_2_ L^−1^/ΔpH, [52]) and calculated from the oxygen carrying capacity and the maximum slope of a fitted pH oxygen-saturation curve. The calculation of putative *in vivo* oxygen saturation and oxygen release required knowledge of *in vivo* venous and arterial *P*O_2_ and pH. Venous and arterial haemolymph *P*O_2_ were not determined for *Octopus pallidus*, *Pareledone charcoti* and *Eledone moschata* in this study and were assumed to correspond to haemolymph *P*O_2_ of *Octopus vulgaris*, which showed an arterial *P*O_2_ of 13 kPa and a venous *P*O_2_ of 4 kPa for resting and 1 kPa for exercised specimens under normoxic conditions [50,51]. Arterial and venous haemolymph *P*O_2_ were further assumed to be constant across temperatures. Venous haemolymph pH was inferred from the alpha-stat regression slope determined for *Octopus* pallidus in this study and corresponded to pH of freshly sampled venous haemolymph of *Pareledone sp.,* and *Eledone moschata* (Figure 7). Due to the difficulty in obtaining arterial haemolymph from small sized octopods, arterial haemolymph pH could not be determined and instead assumed to be 0.11 pH units higher than venous pH as reported for *Octopus vulgaris* [50].

Differences between species and experimental temperatures were tested to be significant (*P* < 0.05) using analysis of variance (ANOVA) followed by Tukey’s *post hoc* test. Non-parametric Kruskal Wallis and Mann–Whitney tests were used to compare the cooperativity of oxygenation dependent proton binding among species. Normality and homogeneity of variance were assessed by Kolmogorov–Smirnov and Levene’s tests, respectively. Results were expressed as means and their 95% confidence interval range if not stated otherwise.

## Additional files

Additional file 1:
**GenBank accession numbers of molecular sequences used for phylogenetic analysis.**
Additional file 2:
**Incomplete desaturation of octopus haemocyanin at low temperatures requires correct identification of the zero calibration point.** (A) At 10°C, haemocyanin of e.g. *Octopus pallidus* fails to fully deoxygenate under pure nitrogen gas and very low pH. Deoxygenation only completes when temperatures increase above 10°C, which complicates the determination of the zero calibration point at low temperature measurement. (B) A linear regression between the absorbance at a reference wavelength (421.75 nm) of the first recorded spectrum and the absorbance peak at 348 nm of fully deoxygenated octopus haemocyanin helped to predict the true zero calibration point at low temperatures for low temperature measurements. The reference absorbance signal at 421.75 nm was selected, as the sum of squares of the differences between the predicted and measured zero calibration point across 20 experiments, were lowest at this wavelength.

## Abbreviations

ATP: Adenosine triphosphate
c(Hc): Haemocyanin concentration
CO_2_: Carbon dioxide
CO_2_: Oxygen carrying capacity
*M*O_2_: oxygen consumption rates of *Pareledone charcoti* (*M*O_2_
MW: Molecular weight
NIST: National Institute of Standards and Technology
*P*CO_2_: Carbon dioxide partial pressure
*P*O_2_: Oxygen partial pressure
*P*_50_: *P*O_2_ at which the pigment is half saturated
pH_50_: pH of haemolymph/blood at which the pigment is half saturated
Q_10_: Temperature coefficient
psu: Practical salinity units
MCMC: Monte Carlo Markov Chain
