# Supplementary material for: Blue blood on ice: modulated blood oxygen transport facilitates cold compensation and eurythermy in an Antarctic octopod
Source: Front Zool. 2015 Mar 11;12:6. doi: 10.1186/s12983-015-0097-x (PMC4403823; doi:10.1186/s12983-015-0097-x)
Supplement: Additional file 1: — GenBank accession numbers of molecular sequences used for phylogenetic analysis. [file 12983_2015_97_MOESM1_ESM.docx]

**Additional file 1**

**GenBank accession numbers of molecular sequences used for phylogenetic analysis.**

| **Species** | **16S** | **COI** | **COIII** | **ODH** | ***rhodopsin*** |
| --- | --- | --- | --- | --- | --- |
| *Abdopus aculeatus* | HM104244 | HM104254 | AJ628213 | HM104276 | HM104287 |
| *Adelieledone polymorpha* | EF102194 | EF102173 | EF102153 | EF102134 | EF102113 |
| *Argonauta nodosa* | AY545104 | AF000028 | AJ628206 | AY545117 | AY545166 |
| *Bathypolypus arcticus* | DQ280044 | AF000029 | KP693813 | - | KP693815 |
| *Benthoctopus cf. rigbyae* | HM572160 | HM572184 | HM572201 | - | HM572217 |
| *Callistoctopus ornatus* | GQ900705 | HM104257 | HM104250 | AY616911 | AY616926 |
| *Eledone cirrhosa* | AY616973 | KP693818 | HM104251 | AY616992 | HM104292 |
| *Eledone moschata* | AJ390326 | KP693816 | KP693814 | - | - |
| *Graneledone verrucosa* | AY545111 | EU071449 | EU071462 | AY545129 | EU086517 |
| *Japetella diaphana* | AJ252766 | AY545192 | EU071453 | AY545130 | AY545179 |
| *Megaleledone setebos* | EF102195 | EF102174 | EF102154 | EU071425 | EF102114 |
| *Octopus pallidus* | AJ252754 | KP693817 | AJ628236 | - | - |
| *Octopus vulgaris* | EF016336 | HM104262 | AJ616311 | HM104284 | HM104297 |
| *Opisthoteuthis massyae* | AY545103 | AY545187 | EU071451 | AY545116 | AY545165 |
| *Pareledone aurata* | EF102198 | EF102177 | EF102157 | EF102136 | EF102118 |
| *Pareledone charcoti* | EF102196 | KP693819 | EF102155 | EF102135 | EF102115 |
| *Pareledone turqueti* | EF102213 | EF102192 | EF102171 | EF102151 | EF102132 |
| *Thaumeledone rotunda* | EU071432 | EU071445 | EU071456 | EU071426 | EU086512 |
| *Vampyroteuthis infernalis* | AY545101 | AF000071 | GU288521 | AY545114 | AY545163 |
