# Supplementary figures and images for: Blue blood on ice: modulated blood oxygen transport facilitates cold compensation and eurythermy in an Antarctic octopod
Source: Front Zool. 2015 Mar 11;12:6. doi: 10.1186/s12983-015-0097-x (PMC4403823; doi:10.1186/s12983-015-0097-x)

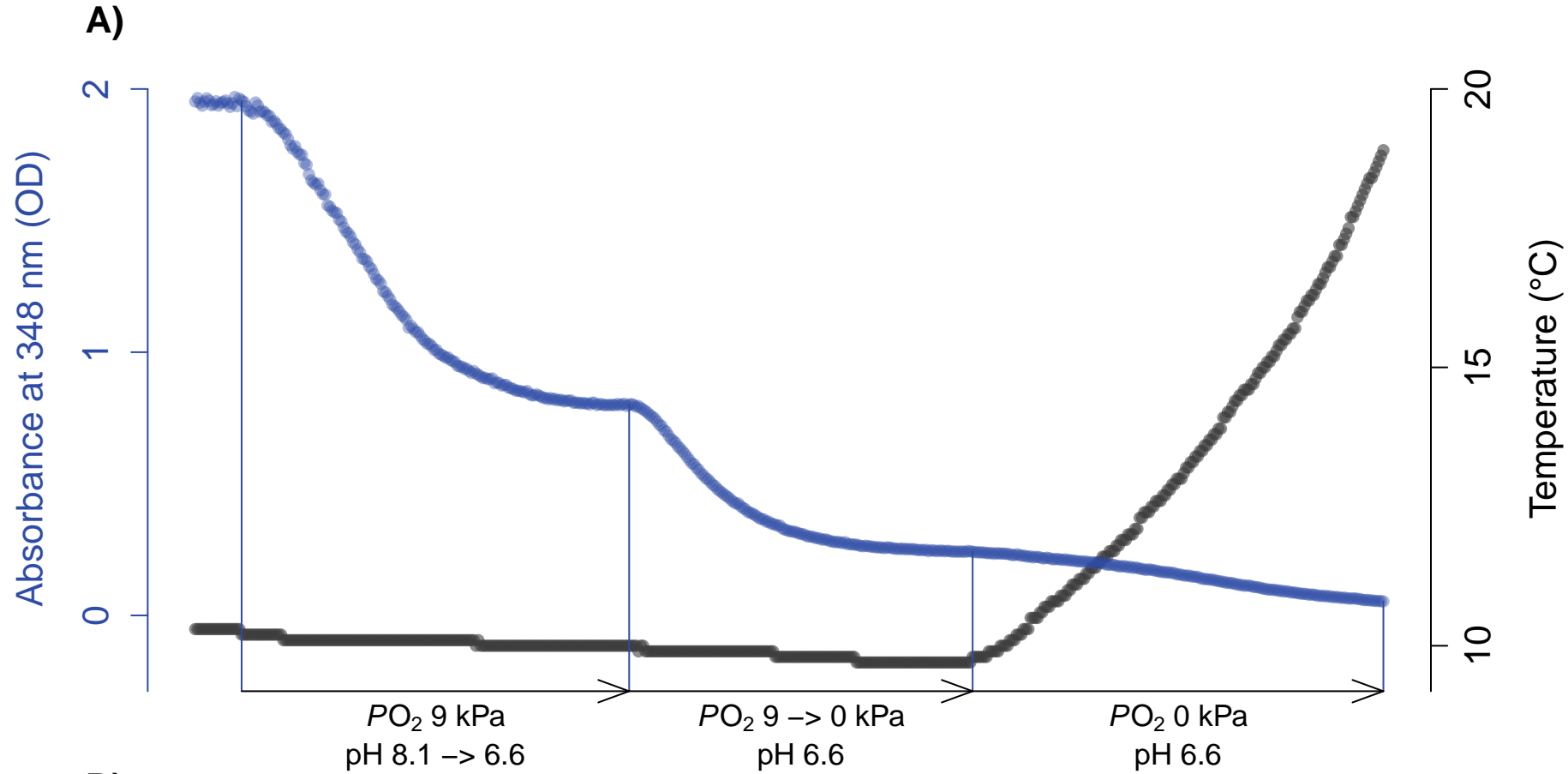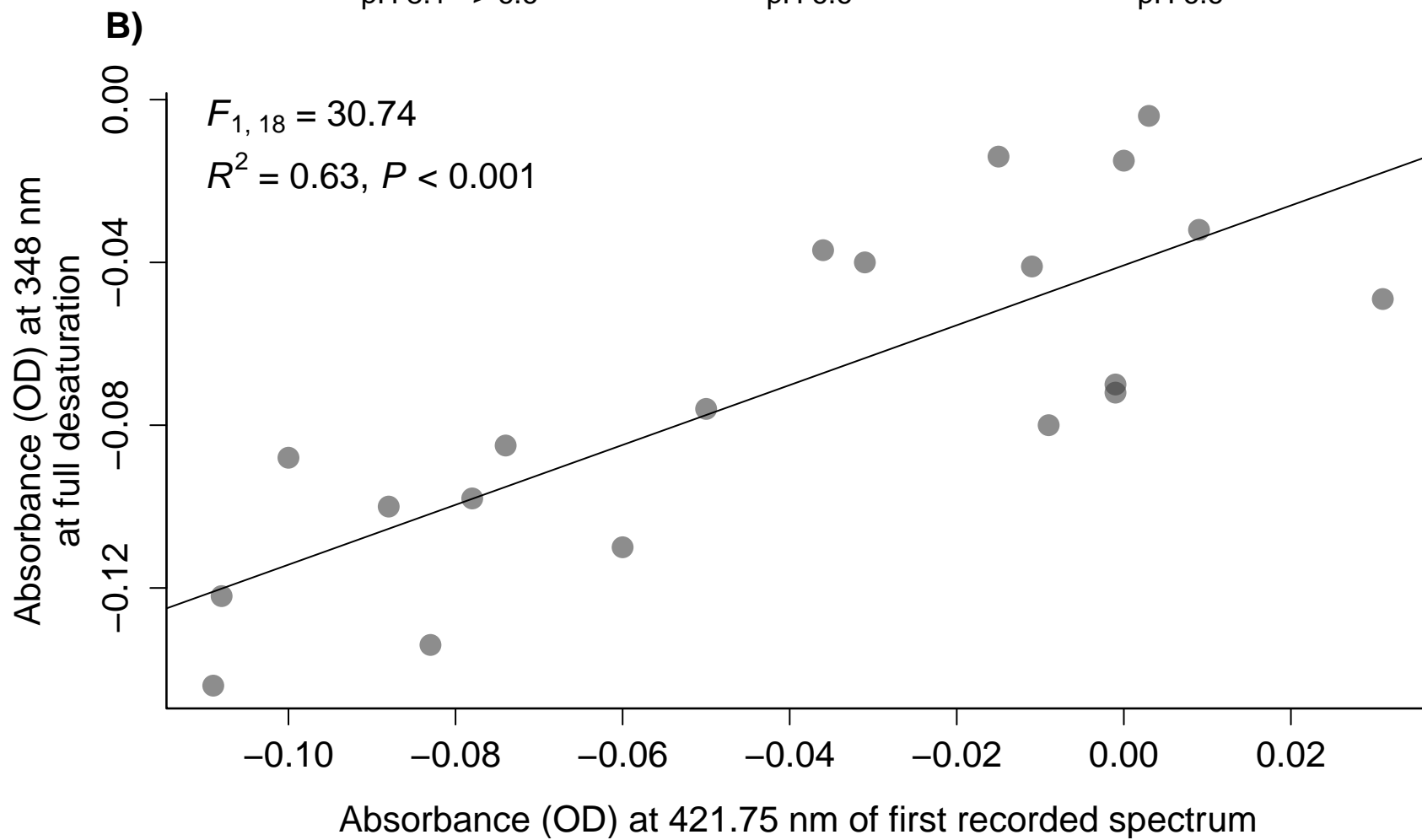

Supplement: Additional file 2: — Incomplete desaturation of octopus haemocyanin at low temperatures requires correct identification of the zero calibration point. (A) At 10°C, haemocyanin of e.g. Octopus pallidus fails to fully deoxygenate under pure nitrogen gas and very low pH. Deoxygenation only completes when temperatures increase above 10°C, which complicates the determination of the zero calibration point at low temperature measurement. (B) A linear regression between the absorbance at a reference wavelength (421.75 nm) of the first recorded spectrum and the absorbance peak at 348 nm of fully deoxygenated octopus haemocyanin helped to predict the true zero calibration point at low temperatures for low temperature measurements. The reference absorbance signal at 421.75 nm was selected, as the sum of squares of the differences between the predicted and measured zero calibration point across 20 experiments, were lowest at this wavelength. [file 12983_2015_97_MOESM2_ESM.pdf]
